# Bladder stone caused by misplaced intrauterine contraceptive device: a case report

**DOI:** 10.11604/pamj.2022.42.143.35362

**Published:** 2022-06-22

**Authors:** Boniface Uji Ago, Glen Enakirerhi

**Affiliations:** 1 Department of Obstetrics and Gynaecology, University of Calabar, Calabar, Nigeria,; 2 Department of Urology, University of Calabar Teaching Hospital, Calabar, Nigeria

**Keywords:** Bladder stone, intrauterine contraceptive device, case report

## Abstract

Intrauterine Contraceptive Device (IUCD) when placed in the uterine cavity is used for contraception or treatment of intrauterine adhesion, but it has become a cause of unintended bladder stone due to wrongful placement or migration. It may cause blood in urine and painful urination. Pelvic ultrasound and X-ray were used to make the diagnosis of the bladder stone with the embedded IUCD, which was removed by open vesicolithotomy through a Pfannenstiel suprapubic incision. Cases such as this are highly preventable if post procedure and routine annual pelvic ultrasonography are emphasized as standard practice following IUCD insertions.

## Introduction

Bladder stones or calculi are hard, calcified mineral materials of uric acid, calcium oxalate, calcium phosphate, ammonium urate, cysteine, and many others [1]. In developing countries, urinary stones have been reported in about 25% of the population [1], but uterine perforation due to IUCD occurs in about 1.6 per 1000 insertions while the IUCD in the bladder becomes a nidus for stone formation [2]. Common complaints are related to bladder wall irritation and inflammation although 30-85% of patients may be asymptomatic [3]. Doing a pelvic ultrasound scan or X-ray often detects the IUCD especially when its location is not certain [3]. We report a case of a 36-year-old nullipara who had post-abortal IUCD insertion at a private hospital 15 years ago.

## Patient and observation

**Patient information:** a 36-year-old nulliparous woman who had IUCD inserted during a termination of pregnancy in a private hospital in Calabar about 15 years earlier. She was not married at the time of the pregnancy and her parents were against her keeping the pregnancy since she was still in school. She got married 10 years after the abortion and IUCD placement and for the subsequent 5 years had been desirous of pregnancy but to no avail. She complained of lower abdominal pains, dysuria and haematuria, which were intermittent but had increased in severity over the last three months. She had gone to the private doctor (who had place the IUCD) for removal but the doctor informed her that the IUCD was missing after several attempts. She was referred to our facility on the 20^th^ of March 2021.

**Clinical findings:** she looked physically stable, but pale. She had no fever and abdominal examination was unremarkable except for suprapubic tenderness.

**Timeline of current of current episode:** she was admitted on the 20^th^ of March 2021 for evaluation and treatment.

**Figure 1:**
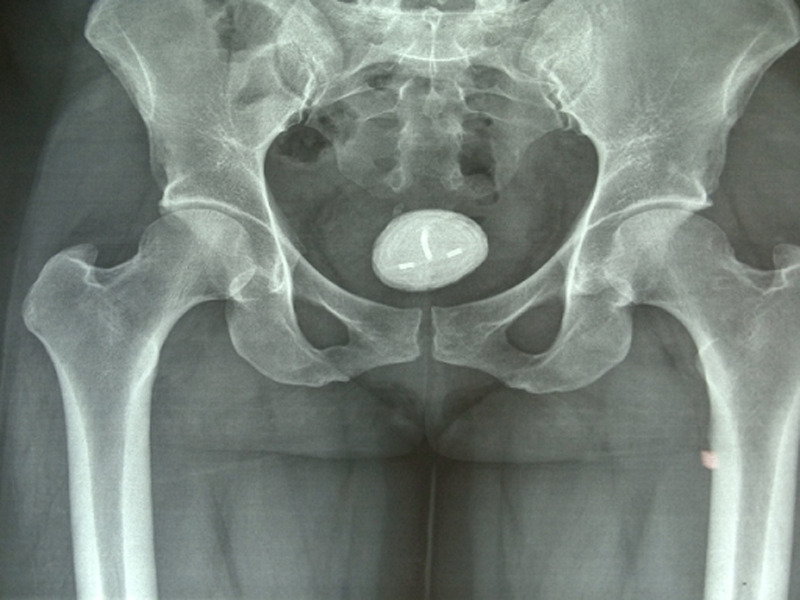
plain X-ray of the pelvis; a calcified bladder mass is noted with the intrauterine contraceptive device embedded in it

**Diagnostic assessment:** urinalysis revealed frank haematuria and both pelvic ultrasound and X-ray revealed that the IUCD was embedded in the urinary bladder. Urine microscopy, culture and sensitivity done prior to surgery showed moderate growth of Escherichia coli sensitive to ceftriaxone for which she was treated. The electrolyte urea and creatinine were within normal limits. Full blood count revealed moderate anaemia for which she was optimized with haematinics before surgery. The X-ray finding is shown in Figure 1.

**Figure 2:**
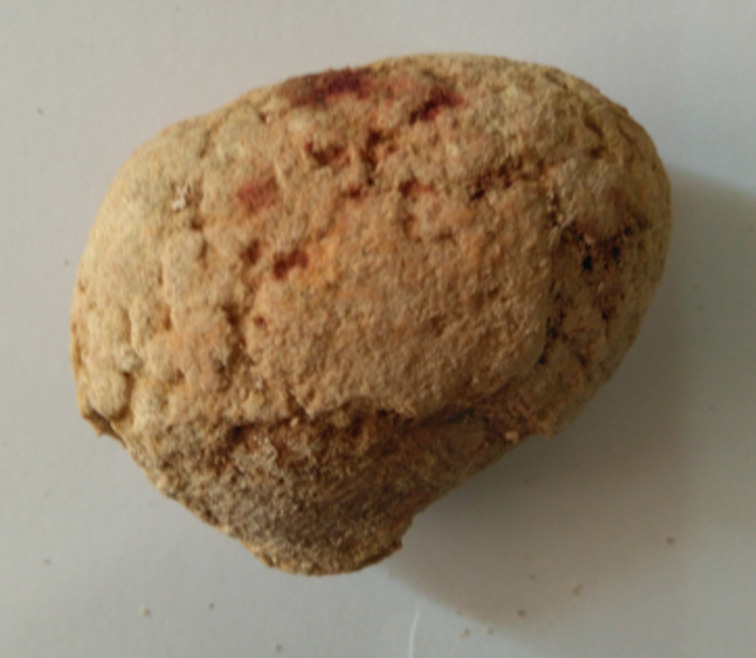
calcified mass removed from the bladder; the mass is shaped like a 6 x 7 cm mango fruit (it is somehow brittle and the surface is irregular)

**Figure 3:**
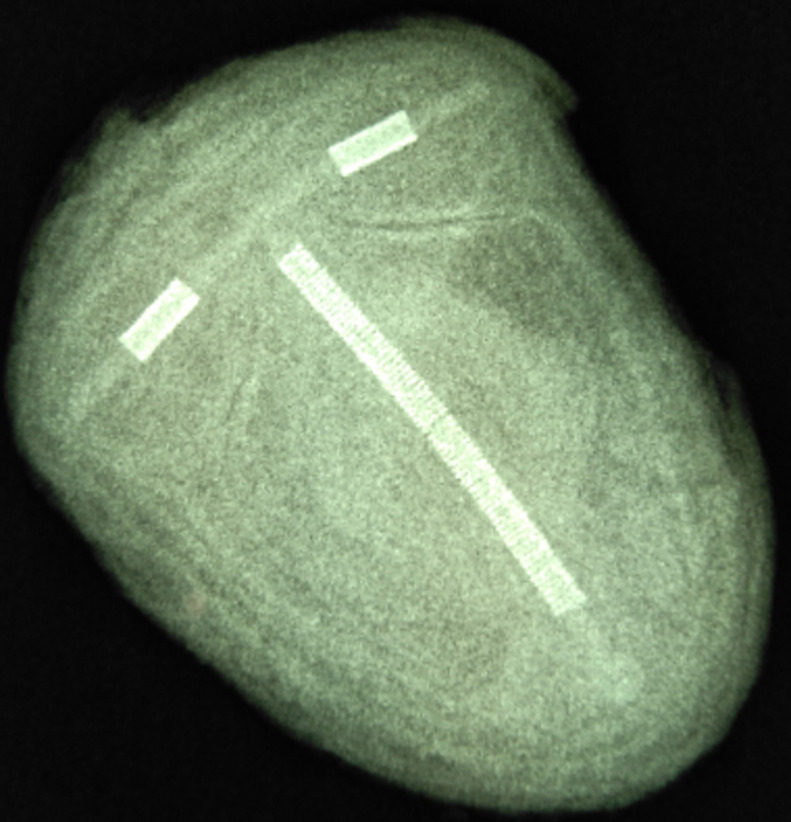
plain X-ray of the bladder stone; this reveals the intrauterine contraceptive device embedded in it

**Therapeutic interventions:** information about the presence of the IUCD in her urinary bladder being the cause of her unwell was discussed with the patient and consent was taken for a transvesical removal. Open vesicolithotomy through a Pfannenstiel suprapubic incision was done and a large bladder stone 6cm by 7cm was removed. The stone is shown in Figure 2. The bladder mucosa though hyperaemic was free of the mass. There were no visible perforations or fistulous tract on the bladder wall. There were no other foreign bodies noted. An X-ray of the bladder stone revealed the presence of IUCD as shown in Figure 3.

**Follow-up and outcomes:** the patient had a stable postoperative period on rectal diclofenac 100mg nocte and oral paracetamol for three days and intravenous ceftriaxone 1 gram daily for five days. She got discharged from the ward to the outpatient clinic after five days but had urethral catheterization for three weeks. Pelvic scan, cystoscopy, and urine microscopy culture and sensitivity done on the third week were unremarkable. She was counselled for infertility evaluation with her husband after three months of recovery.

**Patient perspective:** “I have really suffered because of the abortion I had 15 years ago. I did not know myself when I got pregnant for up to three months. My mother took me to a private doctor who said that he would insert a coil after the abortion to prevent pregnancy until I was married. I did not know the coil shifted to the bladder until I started urinating blood with severe pelvic pains. It was a great relief I had surgery to remove the bladder stone. I thank my team of doctors who have brought me back to good health. My challenge now is how to get pregnant five years after marriage.”

**Informed consent:** this case report met the criteria for waivers of our institutional ethics committee. The patient gave informed consent to the publication of this case for academic and practice changing purposes.

## Discussion

Intrauterine Contraceptive Device (IUCD) inside the urinary bladder is a foreign body capable of irritating the wall and altering the micro and macro environment of the bladder. Several cases of intravesical IUCD have been reported, most of which are due to migration of IUCD after intrauterine insertion [4,5]. Perforation of the uterus, with wrong placement of the IUCD at the time of insertion, and its migration through the anterior uterine wall in close proximity to the bladder, may happen in about 0 - 1.6 per 1000 insertions [2,6]. Post-abortal IUCD insertion may carry an increased risk for this perforation when the uterus is not contracted enough to offer resistance.

Missing IUCD is a known complication of IUCD insertion. When perforation of the uterus is suspected, the WHO recommends that the migrated IUCD should be removed as soon as possible [7]. A common advice for patients after IUCD placement is to feel the thread of the IUCD every morning while bathing. Some patients do not remember to do this, while some are unable to do so yet fail to report to their service providers. Localization of IUCD is often done by ultrasonography and X-ray of the pelvis. This is standard practice and has resulted in the early diagnoses in cases where it is used as part of the diagnostic tool for suspected bladder calculi or missing IUCD [2]. A calcified roundish mass with the IUCD impregnated within on X-ray was usually enough to make a diagnosis [8]. The finding of the X-ray of our patient is shown in Figure 1. It could be argued that if an ultrasound scan was done earlier for our patient, the misplaced IUCD could have been detected and treated appropriately. However, 30 - 85% of patients with displaced or migrated IUCD are asymptomatic [3]. Having post-insertion checks and provision of portable Ultrasound scan services at every Primary Healthcare Center and all centers offering IUCD insertion could help to reduce morbidities associated with this highly effective means of contraception.

Options for the removal of intravesical stones include open vesicolithotomy (cystolithotomy), transurethral or percutaneous cystoscopic removal (cystolitholapaxy) [9], lithotripsy [7] (laser, shock wave, ultrasonic, pneumatic or mechanical), or transvesical laparoendoscopic surgery [10]. The option of treatment depends on the size of the calculi, the facilities and expertise of the surgeon. We opted for open vesicolithotomy (cystolithotomy) because of the large calculi, and because we wanted to remove it intact since the IUCD was completely embedded in the calculi. The size of the stone is similar to that reported by Bolat [11] and De Silva [8] who also did open vesicolithotomy. What is already known is the fact that intravesical IUCDs are a cause of bladder stones. What this report adds is making a case for early recognition and treatment to avert the morbidities associated with this highly preventable problem.

## Conclusion

Several reports of bladder stones caused by the presence of intrauterine contraceptive device in the bladder have confirmed the role of pelvic ultrasonography and X-ray in early diagnosis. Pelvic ultrasonography at least should be part of assessment for women who have IUCD insertion.
